# Reliability of predicting low-burden (≤ 2) positive axillary lymph nodes indicating sentinel lymph node biopsy in primary operable breast cancer — a retrospective comparative study with PET/CT and breast MRI

**DOI:** 10.1186/s12957-023-03297-y

**Published:** 2024-01-06

**Authors:** Chayanee Sae-lim, Wen-Pei Wu, Ming-Che Chang, Hung-Wen Lai, Shu-Tian Chen, Chen-Te Chou, Chiung‑Ying Liao, Hsin-I Huang, Shou-Tung Chen, Dar-Ren Chen, Che-Lun Hung

**Affiliations:** 1 Department of Surgery, Faculty of Medicine, Chulalongkorn University, Bangkok, Thailand; 2 School of Medicine, National Yang Ming Chiao Tung University, Taipei, Taiwan; 3 Department of Biomedical Imaging and Radiological Sciences, National Yang Ming Chiao Tung University, Taipei, Taiwan; 4 Division of General Surgery, Changhua Christian Hospital, Changhua, Taiwan; 5 Comprehensive Breast Cancer Center, Changhua Christian Hospital, Changhua, Taiwan; 6 Endoscopic and Oncoplastic Breast Surgery Center, Changhua Christian Hospital, 135 Nanxiao Street, Changhua, 500 Taiwan; 7 Minimally Invasive Surgery Research Center, Changhua Christian Hospital, Changhua, Taiwan; 8 Department of Radiology, Changhua Christian Hospital, Changhua, Taiwan; 9 Department of Nuclear Medicine, Changhua Christian Hospital, Changhua, Taiwan; 10 Division of Breast Surgery, Yuanlin Christian Hospital, Yuanlin, Taiwan; 11 Kaohsiung Medical University, Kaohsiung, Taiwan; 12 School of Medicine, Chung Shan Medical University, Taichung, Taiwan; 13 Department of Diagnostic Radiology, Chang Gung Memorial Hospital, Chiayi Branch, Chiayi, Taiwan; 14 Institute of Biomedical Informatics, National Yang Ming Chiao Tung University, Taipei, 11221 Taiwan; 15 Department of Information Management, National Sun Yat-Sen University, Kaohsiung, Taiwan; 16 Department of Computer Science and Communication Engineering, Providence University, Taichung, Taiwan; 17 Department of Surgery, National Yang Ming Chiao Tung University, Taipei, Taiwan

**Keywords:** Axillary lymph node, Axillary staging, Breast cancer, Magnetic resonance imaging, Positron emission tomography/computed tomography, Sentinel lymph node biopsy (SLNB), Axillary lymph node disease burden

## Abstract

**Background:**

Sentinel lymph node biopsy (SLNB) is the standard of care for axillary staging in early breast cancer patients with low-burden axillary metastasis (≤ 2 positive nodes). This study aimed to determine the diagnostic performances of 18F-fluorodeoxyglucose (FDG) positron emission tomography/computed tomography (PET/CT) and breast magnetic resonance imaging in detecting axillary lymph node (ALN) metastases and the reliability to predict ALN burden.

**Methods:**

A total of 275 patients with primary operable breast cancer receiving preoperative PET/CT and upfront surgery from January 2001 to December 2022 in a single institution were enrolled. A total of 244 (88.7%) of them also received breast MRI. The sensitivity, specificity, positive predictive value (PPV), negative predictive value (NPV), and accuracy of PET/CT and breast MRI were assessed. The predictive values to determine ALN burden were evaluated using radio-histopathological concordance.

**Results:**

PET/CT demonstrated a sensitivity of 53.4%, specificity of 82.1%, PPV of 65.5%, NPV of 73.5%, and accuracy of 70.9% for detecting ALN metastasis, and the corresponding values for MRI were 71.8%, 67.8%, 56%, 80.8%, and 69.2%, respectively. Combining PET/CT and MRI showed a significantly higher PPV than MRI (72.7% vs 56% for MRI alone, *p* = 0.037) and a significantly higher NPV than PET/CT (84% vs 73.5% for PET/CT alone, *p* = 0.041). For predicting low-burden axillary metastasis (1–2 positive nodes), the PPVs were 35.9% for PET/CT, 36.7% for MRI, and 55% for combined PET/CT and MRI. Regarding patients with 0–2 positive ALNs in imaging, who were indicated for SLNB, the predictive correctness was 96.1% for combined PET/CT and MRI, 95.7% for MRI alone, and 88.6% for PET/CT alone.

**Conclusions:**

PET/CT and breast MRI exhibit high predictive values for identifying low-burden axillary metastasis in patients with operable breast cancer with ≦ 2 positive ALNs on imaging.

**Supplementary Information:**

The online version contains supplementary material available at 10.1186/s12957-023-03297-y.

## Introduction

Sentinel lymph node biopsy (SLNB) has become the mainstay of axillary surgical staging in breast cancer patients with clinically negative (cN0) axillary lymph nodes (ALNs); it results in less morbidity than axillary lymph node dissection (ALND) [1]. The landmark ACOSOG Z0011 trial [2, 3] demonstrated that among patients with T1/T2 primary breast cancer who had no palpable axillary lymph node involvement and underwent breast-conserving surgery with SLNB, the presence of only one or two metastatic sentinel lymph nodes (SLNs) did not compromise overall survival when treated with adjuvant axillary radiotherapy (ART) instead of completion ALND. Furthermore, the AMAROS study [4, 5], which included patients who received a total mastectomy, also reported that SLNB with adjunct ART was noninferior to ALND in terms of survival and locoregional control. These pivotal trials supported that omitting ALND in patients with 1–2 (low burden) positive SLNs followed by ART did not only offer comparable oncological outcomes but also reduced the risk of breast cancer-related lymphedema (BCLE) and improved quality of life (QoL) [3, 6].

As a result, since 2019, the National Comprehensive Cancer Network (NCCN) guidelines for breast cancer [7] have suggested the role of SLNB not only for clinically node-negative patients but also for those with low-burden ALN involvement (≤ 2 positive nodes) diagnosed by imaging or needle biopsy. However, the appropriate imaging modality to determine the “number” of positive ALNs remains a subject of debate. Clinical examination and mammography were not considered as appropriate imaging tools for identifying ALN metastasis. Preoperative ultrasound has an important role in the determination of the pretherapeutic ALN status in patients with newly diagnosed breast cancer; however, it is operator dependent and may have limitations in assessing ALN status, especially in patients with large or dense breasts [8–12].

Magnetic resonance imaging has excellent soft tissue contrast and can detect small metastatic deposits in the lymph nodes that may be missed on an ultrasound [13]. Positron emission tomography (PET), on the other hand, can detect metastases based on metabolic activity, which may be more sensitive than anatomical imaging modalities like ultrasound and MRI [14].

In this study, we aimed to evaluate the diagnostic performances of PET/CT, MRI, and combined PET/CT and MRI in detecting ALN metastasis and predicting ALN burden, including low-burden axillary metastasis (≤ 2 positive lymph nodes), which had not been previously explored.

## Materials and methods

### Study design and populations

**Fig. 1:**
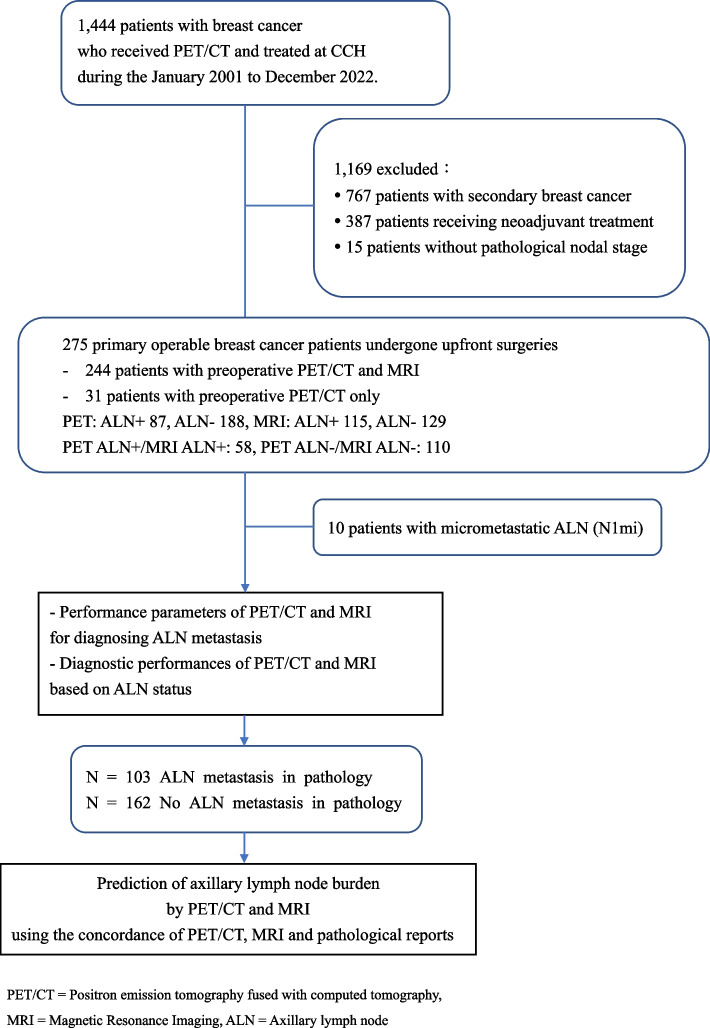
Flow chart of study design

This study included primary operable breast cancer patients who received preoperative PET/CT (with or without breast MRI) for staging and underwent an upfront therapeutic surgery at Changhua Christian Hospital (CCH), a tertiary medical center in Taiwan, from January 2001 to December 2022. The exclusion criteria were secondary breast cancer, receiving neoadjuvant chemotherapy, and no pathological report of axillary nodal status. In our center, PET/CT is typically used for staging advanced disease (beyond stage IIIA) [7] or in patients with aggressive subtypes such as HER-2 overexpression or triple-negative breast cancer (TNBC), which may result in a higher risk of distant metastasis [15]. MRI is commonly employed as a preoperative evaluation tool, providing insights into tumor size, multifocality, multicentric lesions, lymph node status, and the condition of the contralateral breast, especially in patients with the risk associated with inherited breast cancer [7]. The clinicopathological characteristics of patients, including age, tumor size and location, staging, MRI and PET/CT results, surgical procedures, histology and grading, molecular subtypes, and ALN status, were retrieved. All data were collected by specially trained nurses through chart review and subsequently confirmed by the principal investigator (H. W. L.). Ethical approval for the study was obtained from the Institutional Review Board of CCH (CCH IRB No. 230307) and granted a waiver of informed consent. Processes of enrollment and data retrieval were demonstrated in Fig. 1.

### Outcome measures

The primary endpoints of our study were the diagnostic capacities of PET/CT, MRI, and combined PET/CT and MRI in detecting ALN metastasis. Additionally, we conducted a subgroup analysis based on the N staging (N0, N1, N2, N3) [16] to evaluate the performance of these diagnostic tools. Our primary endpoints were determined based on patients with macrometastatic ALNs (tumor deposits measure > 2 mm in the largest dimension) [16] because small tumor deposits observed in SLNB, such as “isolated tumor cells” (N0(i +), tumor deposits measure ≤ 0.2 mm in the largest dimension or have at most 200 cells) [16], and micrometastasis (N1mi, tumor deposits measure > 0.2 mm and ≤ 2 mm in the largest dimension) [16], generally do not necessitate complete ALND [17]. Additionally, including these smaller deposits may introduce potential interference in the evaluation of imaging performance parameters. However, an additional analysis was conducted, incorporating N1mi cases, to investigate potential variations in imaging performance when this specific patient subgroup was included in the assessment.

**Fig. 2:**
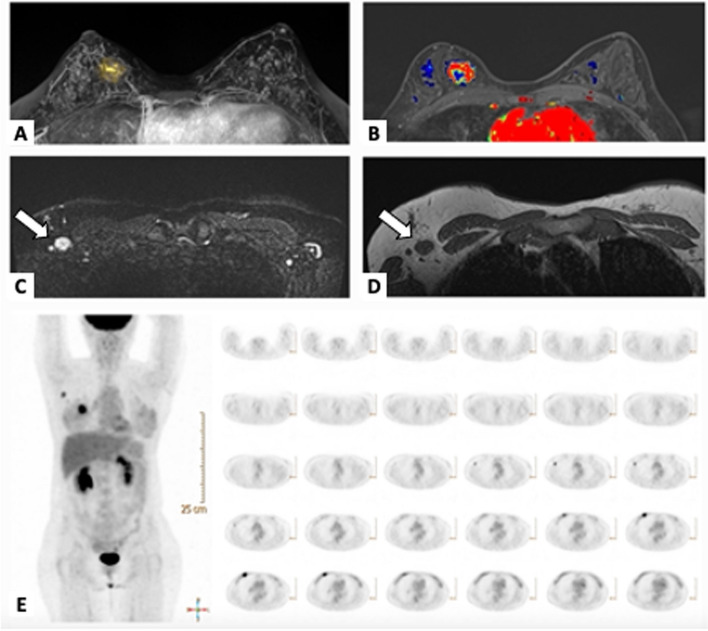
Patient with a concordance between imaging results and pathological results. A 51-year-old female patient with invasive ductal carcinoma in the upper inner quadrant of the right breast. **A**–**B** Breast MRI depicted the round, heterogenous enhancing tumor, between 12 and 2 o’clock, middle-posterior, and 1/3 in depth, approximately 3.2 cm in assessment. T2WI and T1WI images showed a level 1 node (**C**–**D**, arrow). PET/CT showed intense FDG hypermetabolism in the right breast (*SUV*: 7.5 on early images) (**E**) suggesting right breast malignancy and a nodular FDG hypermetabolism in the right axilla (*SUV*: 3.8), suggesting right axillary lymphadenopathy. The final surgical pathology showed invasive ductal carcinoma with a metastatic lymph node (1/23)

**Fig. 3:**
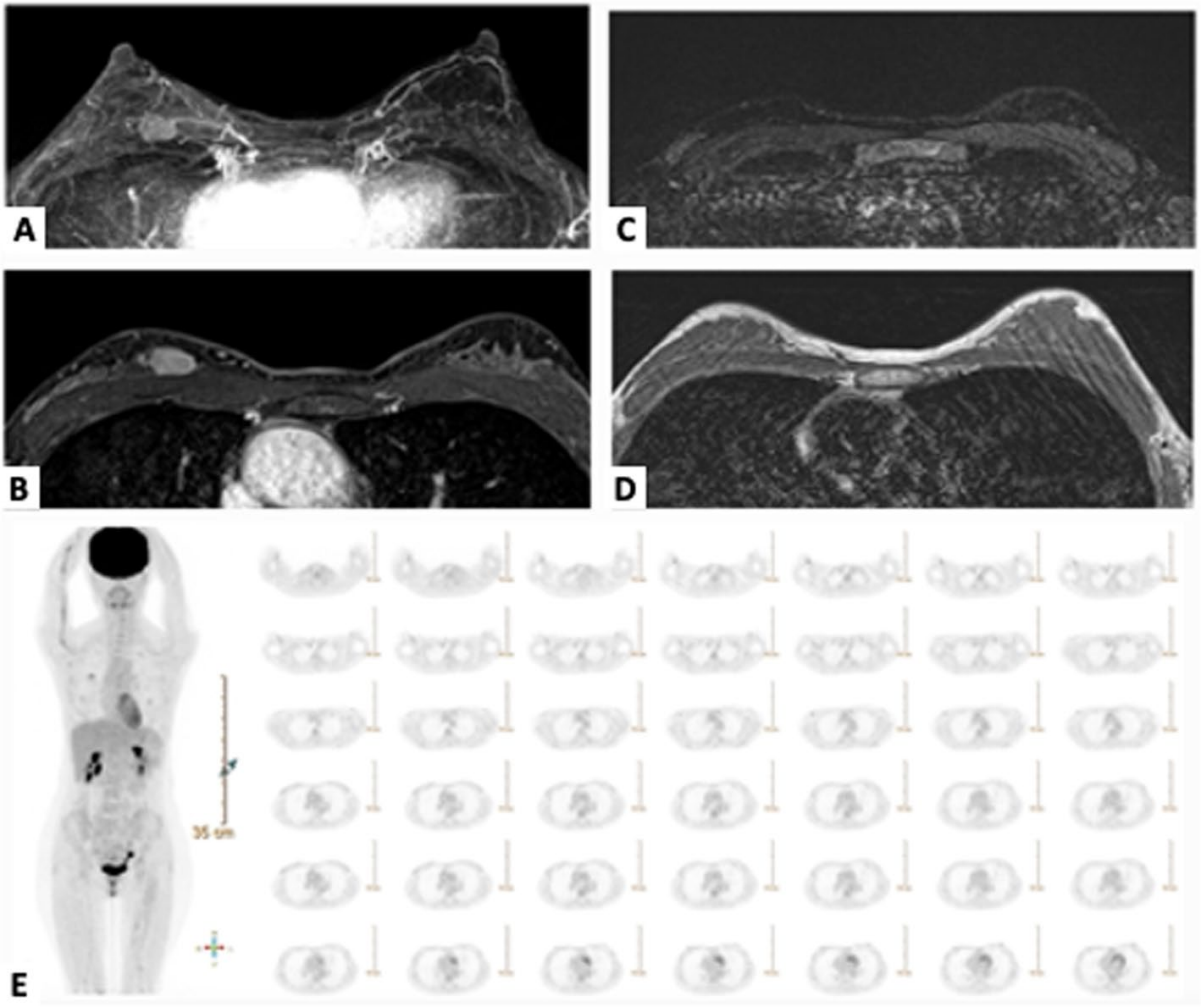
Patient with a discordance between imaging results and pathological results. A 48-year-old female patient with invasive ductal carcinoma in the right breast. **A**–**B** Breast MRI showed a 2-cm round mass. **C**–**D** Axial T2WI and T1WI images showed no enlarged lymph nodes. **E** PET/CT demonstrated intense FDG hypermetabolism in the right breast (*SUV*: 2.29/2.81 on early/delayed images), suggesting breast malignancy and a nodular FDG hypermetabolism in the right axilla (*SUV*: 1.68/1.82), suggesting axillary lymphadenopathy. The final pathological report revealed invasive ductal carcinoma with three metastatic lymph nodes (3/19)

Furthermore, our secondary endpoints were the predictive values of PET/CT, MRI, and combined PET/CT and MRI to predict ALN burden in five groups, including patients with 0, 1, 2, 1–2 (low burden), and 0–2 (indicator for SLNB) [7] positive ALNs on imaging. These predictive values were determined by concordance rates of the number of positive ALNs in each imaging study and the number of macrometastatic ALNs in a pathological examination. Examples of radio-histopathologic concordant and discordant cases were demonstrated in Figs. 2 and 3.

### Image acquisition and interpretation

#### ^18^F-fluorodeoxyglucose positron emission tomography fused with CT (PET/CT)

Before performing PET/CT, patients were required to fast for 4–6 h. Blood glucose levels were checked to ensure a glycemic range below 150 mg/dL. Then patients received an intravenous injection of ^18^F-FDG at 370 MBq. After 60 min of ^18^F-FDG administration, an FDG-PET scan from skull base to mid-thigh was performed using the Gemini GXL 16 PET/CT system, Philips Healthcare. A non-contrast, low-dose CT scan from the skull vertex to mid-thigh was conducted for attenuation correction and anatomical localization. The images were reviewed by an experienced nuclear medicine specialist (M. C. C.) with 12 years of experience in FDG-PET/CT.

The diagnosis of axillary lymph node metastases was considered positive if areas in the axillary region accumulated greater FDG than the surrounding background tissue. The number, region, clinical node staging category, and FDG avidity (SUVmax, defined as the peak standardized uptake value (SUV) in the pixel with the highest count within the region of interest) of suspicious axillary lymph nodes were recorded. The lymph nodes’ morphological size was not used in the final PET/CT criteria.

#### Magnetic resonance imaging

The MRI was performed with a Siemens (Verio) 3.0 Tesla magnet. All patients were imaged in the prone position with both breasts placed into a dedicated 16-channel breast coil. Our standardized breast MRI protocol was reported in a previous study [18]; it includes axial T1-weighted images without fat suppression, axial fat-suppressed T2-weighted images, axial diffusion-weighted images (DWI) and apparent diffusion coefficient (ADC) maps that were created based on DWI sequences, and dynamic fat-suppressed 3D T1-weighted images with one acquisition before contrast injection. All breast MRI data were evaluated by an experienced, board-certified breast radiologist (WPW) with more than 7 years of experience reading breast images.

For the ALN assessment, the criteria used to distinguish nonmetastatic ALNs from metastatic ALNs included size and morphology. A lymph node was considered suspicious for metastasis if it had at least one of the following characteristics: a round or macro-lobulated shape, the absence of fatty hilum, and a cortical thickness greater than 3 mm [18].

#### Combined PET/CT and MRI

To interpret reports from combined PET/CT and MRI, we defined a result to be positive when both modalities detected a suspicious ALN. On the contrary, a negative result from combined PET/CT and MRI was defined as neither PET/CT nor MRI showed evidence of a suspicious ALN.

###  Axillary surgery and radio-histopathologic correlations

Before the breast surgery, a conventional SLNB was performed via an axillary incision with the dual tracers methylene blue and colloidal human serum albumin labeled with technetium-99 m (Tc-99 m). After sentinel nodes were retrieved, they were subjected to frozen section analysis and hematoxylin and eosin staining. If macrometastases were found in the frozen section and met the criteria for ALND [2–5, 7], a conventional ALND was continued in the same operation. The remaining portions of SLNB samples and resected breast tissue were subsequently submitted for permanent sectioning and immunohistochemistry assays.

Imaging performances of PET/CT, MRI, and combined PET/CT and MRI were evaluated through the sensitivity, specificity, positive predictive value (PPV), negative predictive value (NPV), and accuracy, which were calculated by comparing the concordance between preoperative imaging reports and postoperative pathologic ALN statuses. The final histopathological findings obtained from SLNB or ALND were used as the reference standard in our study. Each ALN was assessed for metastasis; then the number of metastatic ALNs was reported. Tumor and nodal stages followed the TNM staging proposed in the AJCC 7th Edition [19].

### Statistical analysis

Patient characteristics were compared using the *χ*^2^ test for categorical variables and the *t*-test for continuous variables. Sensitivity, specificity, positive predictive value (PPV), and negative predictive value (NPV) were computed using McNemar’s test. The differences in diagnostic performance factors were compared using *χ*^2^ analyses or the Kruskal–Wallis test for a non-normal distribution. A *p*-value of less than 0.05 was considered statistically significant. All statistical analyses were performed on a personal computer with the statistical package SPSS for Windows (Version 22.0, SPSS, Chicago).

## Results

### Patient characteristics

In this retrospective study conducted at CCH, a total of 1444 consecutive patients with newly diagnosed breast cancer who underwent preoperative PET/CT (with or without breast MRI) for staging and upfront surgery from January 2001 to December 2022 were reviewed. After excluding 767 patients with secondary breast cancer, 387 patients who received neoadjuvant systemic therapy, and 15 patients with no pathological report of axillary lymph node (ALN) status, 275 women were enrolled in the study. Among this population, 244 of them underwent both preoperative PET/CT and breast MRI, while the remaining 31 patients received PET/CT alone (Fig. 1).

The mean age of the patients was 57.5 ± 12.7 years in the PET/CT group (*N* = 275) and 56.7 ± 12.5 years in the MRI group (*N* = 244) (*p* = 0.71). Of the enrolled patients, 86 (31.3%, 86/275) had positive ALNs in their PET/CT reports, which showed low-burden axillary metastasis in 64 (23.3%) patients (1 positive ALN: 41 patients, 2 positive ALNs: 23 patients). While 115 (47.1%, 115/244) patients had positive ALNs in their MRI reports, 60 (24.6%) of them had low-burden axillary metastasis (1 positive ALN: 40 patients, 2 positive ALNs: 20 patients). There was no significant difference in the techniques of axillary management between these two groups (*p* = 0.69), with SLNB being the most common approach (74.2% in the PET/CT group and 83.9% in the MRI group). Among them, complete ALNDs conducting after indications of macrometastases from SLNBs were reported in 14.6% of the PET/CT group and 19.4% of the MRI group.

The mean numbers of harvested ALNs were not significantly different between the two groups, with 6.5 ± 6.4 ALNs in the PET/CT group and 6.3 ± 6.3 ALNs in the MRI group (*p* = 0.94). The number of metastatic ALNs was also comparable, with 1.4 ± 3.2 metastatic ALNs in the PET/CT group and 1.3 ± 3.3 metastatic ALNs in the MRI group (*p* = 0.85). Among 275 patients in the PET/CT group, 162 (58.9%) of them had no ALN metastasis (pN0), and 113 patients (41.4%) demonstrated ALN metastasis in pathological results, which were 10 patients with pN1mi (3.6%), 72 patients with pN1 (26.2%), 22 patients with pN2 (8%), and 9 patients with pN3 (3.3%). Proportions of pathological N stages in MRI groups were not significantly different from those in the PET/CT group (*p* = 0.88). Characteristics of the patients in the PET/CT and MRI groups were demonstrated in Table 1.

**Table 1:** Patient characteristics

**Characteristics**	**PET/CT **^**a**^ ***N* = 275** **(244 with MRI,** **31 PET/CT alone)**	**MRI**^**b**^ ***N* = 244** **(with PET/CT)**	***p*-value**
**Age, years (mean ± SD)**	57.5 ± 12.8	56.7 ± 12.5	0.71
**Location, *N*(%)**			0.99
Right	127 (46.2)	112 (45.9)	
Left	148 (53.8)	132 (54.1)	
**Tumor size on MRI, cm (mean ± SD)**	3.5 ± 1.8	3.5 ± 1.8	-
**ALN positive in PET report, *N*(%)**			-
Yes	86 (31.3)	-	
No	189 (68.7)	-	
**ALN positive in MRI report, *N*(%)**	*NA* = 31		-
Yes	115 (47.1)	115 (47.1)	
No	129 (52.9)	129 (52.9)	
**Low-burden positive ALN, *N*(%)**			0.85
1 Lymph node	41 (14.9)	40 (16.4)	
2 Lymph nodes	23 (8.4)	20 (8.2)	
**Breast surgery, *N*(%)**			0.78
E-BCS	31 (11.3)	31 (12.7)	
E-NSM/SSM	14 (5.1)	14 (5.7)	
R-NSM	15 (5.5)	15 (6.2)	
C-BCS	98 (35.6)	94 (38.5)	
C-TM/SSM/NSM	117 (42.6)	90 (36.9)	
**Axillary surgery, *N*(%)**			0.69
SLNB	164 (59.6)	163 (66.8)	
SLNB + ALND	40 (14.6)	40 (16.5)	
ALND	71 (25.8)	39 (16.1)	
**Number of harvested ALNs (mean ± SD)**	6.5 ± 6.4	6.3 ± 6.3	0.94
**Number of metastatic ALNs (mean ± SD)**	1.4 ± 3.2	1.3 ± 3.3	0.75
**Pathologic T stage, *N*(%) (*NA* = 8)**			0.99
Tis	6 (2.3)	6 (2.6)	
T1	93 (34.8)	84 (35.4)	
T2	137 (51.3)	123 (51.9)	
T3	26 (9.7)	20 (8.4)	
T4	5 (1.9)	4 (1.7)	
**Pathologic N stage, *N*(%)**			0.88
N0	162 (58.9)	149 (61)	
N1mi	10 (3.6)	10 (4.1)	
N1	72 (26.2)	62 (25.4)	
N2	22 (8)	15 (6.2)	
N3	9 (3.3)	8 (3.3)	
**Pathologic stage, *N*(%) (*NA* = 8)**			0.94
0	4 (1.5)	4 (1.6)	
I	58 (21.1)	53 (21.7)	
II	158 (57.5)	142 (58.2)	
III	36 (13.5)	32 (13.1)	
IV	11 (4.1)	6 (2.4)	
**Histological type, *N*(%)**			0.99
DCIS	6 (2.2)	6 (2.4)	
IDC	232 (84.3)	206 (84.4)	
ILC	18 (6.6)	16 (6.6)	
Others^c^	19 (6.9)	16 (6.6)	
**Grade, *N*(%) (*NA* = 10)**			0.99
I	52 (19.6)	47 (20)	
II	144 (54.3)	125 (53.2)	
III	69 (26.1)	63 (26.8)	
**Molecular subtype, *N*(%) (*NA* = 23)**			0.93
Luminal A	92 (36.6)	83 (36.2)	
Luminal B1	67 (26.6)	62 (27.1)	
Luminal B2	36 (14.2)	34 (14.9)	
HER-2	31 (12.3)	27 (11.8)	
Triple negative	26 (10.3)	23 (10)	

*MRI* magnetic resonance imaging, *ALN* axillary lymph node, *E-BCS* endoscopic-assisted breast conserving surgery, *E-NSM/SSM* endoscopic-assisted nipple/skin-sparing mastectomy, *R-NSM* robotic-assisted nipple-sparing mastectomy, *C-BCS* conventional breast-conserving surgery, *C-TM/SSM/NSM* conventional total mastectomy and nipple-/skin-sparing mastectomy, *SLNB* sentinel lymph node biopsy, *SLNB* + *ALND* sentinel lymph node biopsy followed by axillary lymph node dissection in the same operation, *ALND* axillary lymph node dissection, *DCIS* ductal carcinoma in situ, *IDC* invasive ductal carcinoma, *ILC* invasive lobular carcinoma, *NA* not analyzed

^a^Of 275 patients who underwent preoperative PET/CT, 31 of them underwent PET/CT only, and the other 244 patients also received preoperative MRI

^b^All 244 patients who received preoperative MRI also underwent preoperative PET/CT

^c^Other pathology includes mucinous carcinoma, papillary carcinoma, metaplastic carcinoma, apocrine carcinoma, neuroendocrine carcinoma

### Performance parameters of PET/CT and MRI for diagnosing ALN metastasis

To minimize the potential interference of small tumor deposits, such as N1mi, which typically do not require complete ALND, we assessed the performance parameters based on 265 patients with macrometastatic ALNs (Table 2). PET/CT demonstrated a sensitivity of 53.4%, specificity of 82.1%, PPV of 65.5%, NPV of 73.5%, and overall accuracy of 70.9% for detecting ALN metastasis. The corresponding values for metastatic ALN detection via MRI were 71.8%, 67.8%, 56%, 80.8%, and 69.2%, respectively. When comparing PET/CT and MRI, MRI showed significantly higher sensitivity (71.8% for MRI vs 53.4% for PET/CT, p(1) = 0.01), while a significantly higher specificity was observed in PET/CT (82.1% for PET/CT vs 67.8% for MRI, p(1) = 0.003). However, there were no significant differences in PPV, NPV, and accuracy.

**Table 2:**
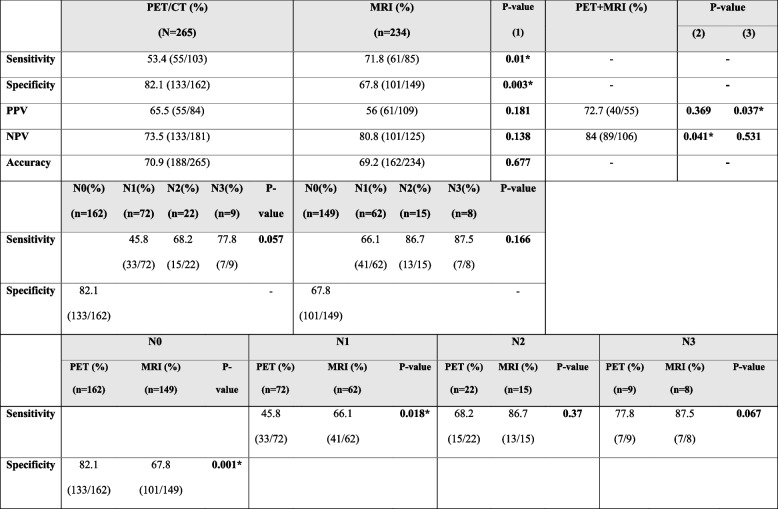
Diagnostic accuracy of MRI and PET/CT for axillary staging

*PET/CT* positron emission tomography fused with computed tomography, *MRI* magnetic resonance imaging, *N0* patients with no metastatic axillary lymph nodes (ALNs), *N1* patients with 1–3 macrometastatic ALNs, *N2* patients with 4–9 macrometastatic ALNs, *N3* patients with at least 10 macrometastatic ALNs [16]

^*^p-value < 0.05, *p*-value (1): PET/CT vs MRI, *p*-value (2): PET/CT vs PET/CT + MRI,*p*-value (3): MRI vs PET/CT + MRI

The combined use of PET/CT and MRI resulted in an improvement in PPV to 72.7%, which was significantly higher than that of MRI alone (56%, p(3) = 0.037) and a significant increase in NPV to 84% when compared to PET/CT alone (73.5%, p(2) = 0.041). The diagnostic performances of all imaging modalities were demonstrated in Table 2.

In addition, we conducted an analysis including 10 patients with N1mi (total of 275 patients, Supplementary Table 1). The results indicated slight decreases in sensitivity and accuracy for both PET/CT and MRI compared to the combined modality performance when focusing only on macrometastasis.

### Diagnostic performances of PET/CT and MRI based on ALN staging

The sensitivity of PET/CT tended to increase from 45.8% in N1 to 77.8% in N3 with borderline statistical significance (*p* = 0.057). The trend of increasing sensitivity following higher pathological N staging was also observed with MRI, which rose from 66.1% in N1 to 87.5% in N3 (*p* = 0.166); however, this difference did not reach statistical significance. Among patients with metastatic ALNs (N1–N3), MRI showed significantly higher sensitivity than PET/CT in patients with N1 (66.1% for MRI vs. 45.8% for PET/CT, *p* = 0.018) and borderline higher sensitivity in patients with N3 (87.5% for MRI vs. 77.8% for PET/CT, *p* = 0.067). The diagnostic parameters of these imaging methods in determining ALN status were demonstrated in Table 2.

For patients with N1mi, the sensitivity of PET/CT was 30%, while that of MRI was 60%. Nevertheless, this difference was not statistically significant (*p* = 0.37). The result was shown in Supplementary Table 1.

### Prediction of ALN burdens

To evaluate the predictive values of ALN burdens, we used the concordance rate between the number of positive ALNs found in imaging reports and the number of metastatic ALNs in pathological results (Table 3). Specifically, among 41 patients with 1 positive ALN in PET/CT, 10 patients were reported to have 1 macrometastatic ALN in pathological results, resulting in a PPV of 24.4% (10/41) for PET/CT to predict 1 metastatic ALN, which was comparable to a PPV of 22.5% (9/40) for MRI (p(1) = 0.841). However, among the 9 patients with 1 positive ALN detected by both PET/CT and MRI, 4 of them demonstrated 1 macrometastatic ALN in their pathological reports, indicating a trend towards an increased PPV of 44.4% (4/9) for the combined PET/CT and MRI results. Despite this improvement, the difference did not reach statistical significance when compared to PET/CT alone (44.4% vs. 24.4%, p(2) = 0.25) or MRI alone (44.4% vs. 22.5%, p(3) = 0.22). A similar trend of increased PPV when combining the PET/CT and MRI results was observed in patients with two positive ALNs.

**Table 3:** Prediction of axillary lymph node (ALN) burden

**Number of positive ALN on imaging**	**PET/CT**	**MRI**	***p*-value** **(1)**	**PET/CT + MRI**	***p*-value** **(2)**	***p*-value** **(3)**
	**NPV (%)**	**NPV (%)**		**NPV (%)**		
**0**	73.5 (133/181)	80.8 (101/125)	0.138	84 (89/106)	0.041*	0.531
	**PPV (%)**	**PPV (%)**		**PPV (%)**		
**1**	24.4 (10/41)	22.5 (9/40)	0.841	44.4 (4/9)	0.25	0.22
**2**	8.7 (3/23)	30 (6/20)	0.263	33.3 (1/3)	0.4	> 0.999
**1–2 (low burden)**	**35.9 (23/64)**	**36.7 (22/60)**	**0.933**	**55 (11/20)**	**0.13**	**0.149**
**0–2 (indicator for SLNB)**	**88.6 (217/245)**	**95.7 (177/185)**	0.008*	**96.1 (173/180)**	0.005*	**0.834**

^*^p-value < 0.05

*p*-value (1): PET/CT vs MRI, *p*-value (2): PET/CT vs PET/CT + MRI, *p*-value (3): MRI vs PET/CT + MRI

In the context of low-burden-positive ALNs in imaging (1–2 ALNs), all imaging modalities exhibited higher PPVs compared to those of only one or two positive ALNs; the PPVs were 35.9% (23/64) for PET/CT, 36.7% (22/60) for MRI, and 55% (11/20) for combined PET/CT and MRI.

Regarding patients with 0–2 positive ALNs in imaging, which were indicated for SLNB, the correctness for predicting 0–2 metastatic ALNs in pathological results was 96.1% (173/180) for combined PET/CT and MRI and by 95.7% (177/185) for MRI alone, which were significantly higher than 88.6% (217/245) for PET/CT alone (96.1% vs 88.6%, p(2) = 0.005, 95.7% vs 88.6%, p(1) = 0.008). The predictive values of all imaging modalities were demonstrated in Table 3.

## Discussions

This retrospective study aimed to investigate the diagnostic performances of PET/CT, MRI, and both modalities combined to determine ALN metastasis and the potential to predict ALN burden, which has become essential in selecting patients for SLNB and was rarely discussed before. After screening 1444 breast cancer patients, a total of 275 primary operable breast cancer patients with preoperative PET/CT who received upfront breast cancer surgeries were enrolled for the current study. Among them, 244 (88.7%) also received breast MRI before surgery, enabling us to compare the diagnostic performances of PET/CT and breast MRI in almost similar groups of patients (Table 1). The sensitivity, specificity, PPV, NPV, and accuracy were 53.4%, 82.1%, 66.5%, 73.5%, and 70.9% for PET/CT, and the corresponding values for MRI were 71.8%, 67.8%, 56%, 80.8%, and 69.2%, respectively (Table 2). These results were consistent with previous literatures, which were summarized in Table 4 [10–12, 20–31].

**Table 4:** Summary of previous studies regarding performance of PET/CT and MRI in diagnosing axillary lymph node metastasis

**Positron emission tomography fused with computed tomography (PET/CT)**								
**Study**	**Journal/year/patients**	**PET/CT drug and dose**	**Diagnostic criteria**	**Sensitivity (%)**	**Specificity (%)**	**PPV (%)**	**NPV (%)**	**Accuracy (%)**
Stadnik et al. [20]	Eur Radiol./2006/10	18F-FDG, 464 ± 56 MBq	NR	100	80	80	100	NR
Riegger et al. [12]	Acta Radiol./2012/90	18F-FDG, 210–360 MBq	Focally increased PET signal	54	89	77	74	75
Choi et al. [21]	J Breast Cancer/2012/154	18F-FDG, 5.55 MBq/kg	Pathologic uptake higher than the liver activity	37.3	95.8	NR	NR	NR
Hwang et al. [11]	J Breast Cancer/2013/349	18F-FDG, 8.1 MBq/kg	Higher level uptake than the background	44.5	94.2	73.2	82.6	81.1
An et al. [10]	Nuklearm-edizin/2014/215	18F-FDG, 370 MBq	Higher level uptake than that of normal background soft tissue and SUVmax	62.7	88.6	77.6	79.1	78.6
Kitajima et al. [22]	Jpn J Radiol/2016/196	18F-FDG, 4.0 MBq/kg	Higher level uptake than the background and SUVmax	55.4	95.8	NR	NR	84.5
Orsari et al. [23]	Anticancer Res./2018/50	18F-FDG, 370–450 MBq	Higher level uptake than the background and SUVmax	87	90	93	82	88
Kutluturk et al. [24]	Niger J Clin Pract. /2019/232	18F‐FDG, 0.1 mg/kg	NR	72.6	77.9	88.8	54	74.1
Assi et al. [25]	Front Oncol./2021/268	18F-FDG, 180–296 MBq	Focal uptake with a strong target-to-background ratio	86.6	63.5	78.9	75	77.6
**Sae-lim et al**	**Current study/275**	**18F-FDG, 370 MBq**	**Higher level uptake than the background and SUVmax**	**51.3**	**82.7**	**67.4**	**70.9**	**69.8**
**Magnetic resonance imaging**								
**Study**	**Journal/year/patients**	**Magnetic field strength**	**Diagnostic criteria**	**Sensitivity (%)**	**Specificity (%)**	**PPV (%)**	**NPV (%)**	**Accuracy (%)**
Hwang et al. [11]	J Breast Cancer/2013/349	1.5 T	Eccentric cortical thickening, irregular or round shape, loss of fatty hilum	47.8	88.7	60.2	82.6	77.9
An et al. [10]	Nuklearm-edizin/2014/215	1.5, 3.0 T	Cortical thickening > 3 mm, obliteration of fatty hilum, mass appearance, regular or round shape	67.5	78	65.9	79.2	74
Kim et al. [26]	Clinical breast cancer/2017/147	3.0 T	Shape, size, the presence of fatty hilum, asymmetrical cortical thickness, ADC value	51.3–59	93.6	74.1–76.7	84.4–86.6	NR
van Nijnatten et al. [27]	Clin Radiol./2018/90	3.0 T	The absence of contrast hyperintensity and absence of an intact nodal border	38–60	89–93	56–64	79–92	NR
Ahn et al. [28]	Radiol Med./2019/74	3.0 T	Short axis > 0.5 cm, cortical thickness > 0.3 cm, eccentric cortical thickening, loss or compression of the fatty hilum	52.9	89.5	60	86.4	NR
Guvenc et al. [29]	The breast journal/2019/85	1.5, 3.0 T	Complete absence of the central fatty hilum, LN short axis > 1 cm, displaced fatty hilum, eccentric cortical thickening, matted LNs, irregular cortex, loss of intensity on T2‐weighted imaging, ADC value	79–83	81–98	65–95	89–93	NR
Ramírez-Galván et al. [30]	Acta Radiol. 2020/44	1.5 T	Cortical thickening, obliteration of fatty hilum, mass appearance, ADC value	66.7	76.7	NR	NR	NR
Kurt et al. [31]	Diagn Interv Radiol./2022/66	1.5 T	Large LN, increased cortex thickness, obliterated hilum	76.7–83.7	69.6–78.3	69.6–86.8	64.3–83.7	NR
**Sae-lim et al**	**Current study/244**	**3.0 T**	**Macrolobulated shape, the absence of fatty hilum, cortical thickness greater than 3 mm**	**70.5**	**67.8**	**58.3**	**78.3**	**68.9**

*PPV* positive predictive value, *NPV* negative predictive value, *NR* not reported, *SUVmax* maximum standardized uptake value, *T* Tesla, *ADC* apparent diffusion coefficient

Our results demonstrated some significant differences in the diagnostic performances of PET/CT, MRI, and combined PET/CT and MRI for detecting ALN metastasis (Table 2). When comparing MRI and PET/CT, MRI showed significantly higher sensitivity (71.8% vs 53.4%, p(1) = 0.01), while PET/CT demonstrated significantly greater specificity than MRI (82.1% vs 67.8%, p(1) = 0.003). Our results align with a previous meta-analysis conducted by Cooper et al. [32], which assessed the capabilities to determine ALN metastasis by PET/CT and MRI and other studies that recognized PET/CT and MRI as important tools in breast cancer staging nowadays [33–39].

The combination of PET/CT and MRI could improve predictive values of each modality in specific patients with concordant results between both PET/CT and MRI (Table 2). Specifically, in patients who had positive ALNs in both PET/CT and MRI results, there was significant improvement of PPV compared to MRI alone (72.7% vs 56%, p(3) = 0.037). On the other hand, patients with negative ALN results in both PET/CT and MRI revealed a significant increase in NPV compared to patients with negative ALN results in PET/CT (84% vs 73.5%, p(2) = 0.041). However, in cases where PET/CT and MRI results were discordant (negative PET/CT and positive MRI or positive PET/CT and negative MRI), the decision regarding surgical management should rely on the performance of a single modality.

As completion ALND generally was not recommended for micrometastasis (N1mi) [16] found in SLNB when appropriate adjuvant therapy was intended [17], the primary endpoints mentioned above were designed to assess only macrometastatic ALNs [16]. However, there were 10 patients with N1mi in our populations, prompting us to conduct an additional analysis that included patients with N1mi (Supplementary Table 1). The results indicated slight decreases in sensitivity and accuracy for both PET/CT and MRI when compared to the performance of both modalities when focusing solely on macrometastasis. These results may be attributed to the sensitivity for detecting N1mi appearing to be lower than that for macrometastasis (N1-3).

Since the results of ACOSOG Z0011 [2], SLNB has become the standard of care for axillary management of breast cancer patients with clinically negative ALN results or low-burden ALN metastases (one or two positive nodes) contained in SLNs. The same trend was observed in results in this study, up to 74.2% (204/275) of patients underwent SLNB, and only 40 of those patients required completion ALND in the same operation. Additionally, since 2019, the NCCN guidelines for breast cancer [7] suggested the role of SLNB not only for clinically negative ALN results but also for low-burden (one or two positive) axillary lymph node involvement diagnosed by imaging or needle biopsy. However, choosing the proper imaging modality to determine ALN burden is still the major challenge.

Regarding the ability to predict a specific number of metastatic ALNs, such as one or two ALNs, the diagnostic performances of PET/CT and MRI exhibited modest predictive values, ranging from 8.7 to 33% (Table 3). When using the criterion of both PET/CT and MRI revealing at least one positive ALN, there was a slight increase in PPV, but these changes did not reach statistical significance (p(2), p(3) ≥ 0.05). However, in patients with 1–2 positive ALNs on imaging, the PPV for determining low-burden (1–2 positive nodes) ALN metastasis appeared to improve to 35.9% for PET/CT, 36.7% for MRI, and 55% for combined PET/CT and MRI.

Furthermore, according to considerations for SLNB given in the NCCN guidelines [7], we focused on patients with ≤ 2 suspicious ALNs on imaging to assess the correctness for predicting 0–2 metastatic ALNs. Our results revealed a substantial improvement in PPV, reaching 96.1% with combined PET/CT and MRI, 95.7% for MRI alone, and 88.6% for PET/CT alone, indicating the potential that these patients may not require ALND [7]. Therefore, our study suggests that MRI and PET/CT provide high predictive values for identifying no ALN metastasis or low-burden ALN metastasis in patients with at most two positive ALNs on these imaging modalities.

The current study has limitations owing to its retrospective design and potential selection biases. Firstly, we excluded patients who received neoadjuvant treatment in order to minimize its impact on pathological axillary staging. Secondly, our study focused on PET/CT and MRI because these modalities allow for objective re-evaluation by doctors, unlike axillary sonography, which is often operator dependent [8–12]. However, we acknowledge the higher cost for PET/CT, which may affect its widespread application. Despite these limitations, our study provided valuable insights of predicting ALN burdens and supported considerations of MRI and PET/CT as imaging modalities to determine the role of SLNB. Additionally, our study provides a foundation for future research, particularly in exploring the application of artificial intelligence and machine learning to enhance the diagnostic performance of PET/CT and MRI for axillary metastasis.

## Conclusion

Our results demonstrated that PET/CT and MRI provide high predictive values for identifying low-burden ALN metastasis in patients with ≤ 2 positive ALNs on imaging, which could have significant implications for the omission of ALND in breast cancer patients.

### Supplementary Information

**Additional file 1: Supplementary Table 1. **Diagnostic parameters of PET/CT and MRI for axillary staging, including micrometastasis (N1mi).

## Data Availability

The datasets used and/or analyzed during the current study are available from the corresponding author (Hung-Wen Lai) on reasonable request.

## Abbreviations

SLNB: Sentinel lymph node biopsy
ALND: Axillary lymph node dissection
ALN: Axillary lymph node
BCLE: Breast cancer-related lymph edema
NCCN: National Comprehensive Cancer Network
SUV: Standardized uptake value
FDG: 18F-fluorodeoxyglucose
PET/CT: Positron emission tomography/computed tomography
MRI: Magnetic resonance imaging
PPV: Positive predictive value
NPV: Negative predictive value
ADC: Apparent diffusion coefficient
DWI: Diffusion-weighted images
ART: Axillary radiotherapy
SLN: Sentinel lymph node
