# Supplementary material for: Reliability of predicting low-burden (≤ 2) positive axillary lymph nodes indicating sentinel lymph node biopsy in primary operable breast cancer — a retrospective comparative study with PET/CT and breast MRI
Source: World J Surg Oncol. 2024 Jan 6;22:12. doi: 10.1186/s12957-023-03297-y (PMC10770957; doi:10.1186/s12957-023-03297-y)
Supplement: Supplementary file 1 — Additional file 1: Supplementary Table 1. Diagnostic parameters of PET/CT and MRI for axillary staging, including micrometastasis (N1mi). [file 12957_2023_3297_MOESM1_ESM.docx]

|  | PET/CT (%)  (n = 275) | | | | | | | | MRI (%)  (n=244) | | | | | | P-value  (1) | | PET+MRI (%)  (n=168) | | | | | | P-value | | |
| --- | --- | --- | --- | --- | --- | --- | --- | --- | --- | --- | --- | --- | --- | --- | --- | --- | --- | --- | --- | --- | --- | --- | --- | --- | --- |
|  |  |  |  |  |  |  |  |  |  |  |  |  |  |  |  |  |  |  |  |  |  |  | **(2)** | | **(3)** |
| Sensitivity | 51.3 (58/113) | | | | | | | | 70.5 (67/95) | | | | | | **0.005*** | | - | | | | | | **-** | | |
| Specificity | 82.1(133/162) | | | | | | | | 67.8 (101/149) | | | | | | **0.002*** | | - | | | | | | **-** | | |
| PPV | 66.7 (58/87) | | | | | | | | 58.3 (67/115) | | | | | | **0.184** | | 74.1(43/58) | | | | | | **0.338** | | **0.04*** |
| NPV | 70.7 (133/188) | | | | | | | | 78.3 (101/129) | | | | | | **0.140** | | 80.9(89/110) | | | | | | **0.052** | | **0.618** |
| Accuracy | 69.5 (191/275) | | | | | | | | 68.9 (168/244) | | | | | | **0.812** | | - | | | | | | **-** | | |
|  | **PET/CT** | | | | | | | | | | | | **MRI** | | | | | | | | | | | | |
|  | **N0(%)**  **(n=162)** | | **N1mi(%)**  **(n=10)** | | **N1(%)**  **(n=72)** | | **N2(%)**  **(n=22)** | | **N3(%)**  **(n=9)** | | **P-value** | | **N0 (%)**  **(n=149)** | | **N1mi (%)**  **(n=10)** | | **N1(%)**  **(n=62)** | **N2 (%)**  **(n=15)** | | | **N3(%)**  **(n=8)** | | **P-value** | | |
| Sensitivity | - | | 30  (3/10) | | 45.8  (33/72) | | 68.2  (15/22) | | 77.8  (7/9) | | **0.055** | | - | | 60  (6/10) | | 66.1  (41/62) | 86.7  (13/15) | | | 87.5  (7/8) | | **0.271** | | |
| Specificity | 82.1  (133/162) | |  | | - | | - | | - | | - | | 67.8  (101/149) | |  | | - | - | | | - | | - | | |
|  | **N0** | | | | | **N1mi** | | | | **N1** | | | | | | **N2** | | | | **N3** | | | | | |
|  | **PET(%)**  **(n=162)** | **MRI(%)**  **(n=149)** | | **P-value** | | **PET(%)**  **(n=10)** | | **MRI(%)**  **(n=10)** | **P-value** | **PET(%)**  **(n=72)** | | **MRI(%)**  **(n=62)** | | **P-value** | | **PET(%)**  **(n=22)** | **MRI(%)**  **(n=15)** | | **P-value** | **PET(%)**  **(n=9)** | | **MRI(%)**  **(n=8)** | | **P-value** | |
| Sensitivity | - | - | | - | | 30  (3/10) | | 60  (6/10) | **0.37** | 45.8  (33/72) | | 66.1  (41/62) | | **0.002*** | | 68.2  (15/22) | 86.7  (13/15) | | **0.37** | 77.8  (7/9) | | 87.5  (7/8) | | **0.067** | |
| Specificity | 82.1  (133/162) | 67.8  (101/149) | | **0.001*** | |  | |  |  | - | | - | | - | | - | - | | - | - | | - | | - | |

**Supplementary Table 1** Diagnostic parameters of PET/CT and/or MRI for axillary staging, including micrometastasis (N1mi)

*P-value <0.05, P-value (1): PET/CT vs MRI, P-value (2): PET/CT vs PET/CT+MRI, P-value (3): MRI vs PET/CT+MRI

PET/CT = positron emission tomography fused with computed tomography, MRI = magnetic resonance imaging,
N0 = patients with no metastatic axillary lymph nodes (ALNs), N1mi = patients whose tumor deposits in ALNs measure >0.2 mm and ≤ 2 mm in the largest dimension, N1 = patients with 1-3 macrometastatic ALNs, N2 = patients with 4-9 macrometastatic ALNs, N3 = patients with at least 10 macrometastatic ALNs^(1)^

1. Hortobagyi G, Connolly J. Breast In: Amin MB, Edge S, Greene F, eds. AJCC Cancer Staging Manual, 8th edn New York, NY: Springer International Publishing. 2016.
